# Direct observation of positron capture process at the positron source of the superKEKB B-factory

**DOI:** 10.1038/s41598-022-22030-5

**Published:** 2022-11-03

**Authors:** Tsuyoshi Suwada

**Affiliations:** 1 Accelerator Laboratory, High Energy Accelerator Research Organization (KEK), Tsukuba, Ibaraki 305-0801 Japan; 2 School of High Energy Accelerator Science, SOKENDAI (The Graduate University for Advanced Studies), Tsukuba, Ibaraki 305-0801 Japan

**Keywords:** Applied physics, Techniques and instrumentation, Particle physics

## Abstract

A direct simultaneous detection of electron ($e^-$) and positron ($e^+$) bunches was successfully performed using wideband beam monitors and a detection system at the $e^+$ capture section of the SuperKEKB B-factory. The time intervals between the secondary-generated $e^-$ and $e^+$ bunches were measured to investigate their dynamical capture process. The results show that the time intervals were measured in the range of 20–280 ps on average, and the line-order switch of the $e^-$ and $e^+$ bunches and their dynamical phase-slip process in the axial direction were clearly observed as a function of the capture phase of accelerating structures. In this report, the dynamical capture and phase-slip process for the $e^-$ and $e^+$ bunches under the two-bunch acceleration scheme is described in detail. This study opens up a new window for direct observation of the $e^-$ and $e^+$ capture process at any present and future $e^+$ sources.

High-intensity positron sources are indispensable in high-energy $e^+e^-$ colliders to achieve the high luminosity required for high-energy physics experiments. A number of excellent reviews of high-intensity $e^+$ sources^1,2^ in high-energy lepton colliders are available, where any interested reader may find detailed reviews and technical challenges of not only advanced and novel $e^+$ sources in future projects but also current conventional $e^+$ sources (see references therein for an extended list).

In conventional $e^+$ sources^3–5^, positrons are produced by impinging high-energy and high-intensity primary electron beams to a target of a high-*Z* material through a $e^+e^-$ pair-production process in an electromagnetic cascade shower. Both the secondary-generated $e^-$ and $e^+$ bunches with approximately equivalent amounts of bunch charges simultaneously emerge from the target back surface through this process. They are immediately captured in the subsequent $e^+$ capture section, in which they are focused with a strong pulsed magnetic field by a flux concentrator (FC) to attain the maximum capture efficiency (called the adiabatic matching device (AMD), see Ref. ^6^ for details). After being focused in the FC, the $e^-$ and $e^+$ bunches are immediately accelerated in multiple accelerator structures embedded in DC solenoid coils. While the $e^-$ bunch is stopped by a beam stopper (BS) after passing the $e^+$ capture section, the positrons are separated from the electrons and further accelerated by subsequent accelerator structures. The $e^+$ intensity can be firstly measured using a beam position monitor (BPM) after the $e^+$ capture section because it has never been measured in the $e^+$ capture section so far.

There are mainly three reasons why the measurement is difficult. Firstly, there is almost no space to install any instrumentation devices in the conventional $e^+$ capture section. Secondly, the radiation environment poses great difficulty in the measurement because the location of the target is close to all the instrumentation devices. Finally, the time interval between the $e^-$ and $e^+$ bunches is very short because they pass almost simultaneously through the capture section with a certain time interval. The typical time interval between them is in the order of 100 ps. These are the reasons why beam parameters have never been measured at the conventional $e^+$ capture section, although they are generally simulated on the basis of beam dynamics in multidimensional transverse and longitudinal phase spaces. It is a challenging task to experimentally verify and elucidate complicated beam dynamics for both positrons and electrons in the $e^+$ capture section in order to fully understand them and to maximize the $e^+$ intensity under an optimized operation condition.

Multidimensional optimizations should be applied to the $e^+$ source to experimentally increase the bunch intensity in a multidimensional parameter space, which is based on electromagnetic fields of accelerating structures and magnetic solenoidal fields in the capture section. Transverse positions, injection angles, and radii of primary electrons impinging on the target are also parameters in the multidimensional parameter space. However, it is generally difficult to find out not the local optimum but the global optimum in such multidimensional optimizations, because no instrumentation devices in the conventional capture section have so far been available. This is because it is difficult to independently measure both the $e^-$ and $e^+$ bunches by a conventional technique. This is the main reason why new wideband beam monitors (WBMs) should be installed in the capture section.

## Results

### Waveform analysis of the pickup signals

The $e^+$ source of the SuperKEKB B-factory and the simultaneous detection system of the $e^+$ and $e^-$ bunches by using two WBMs with four-pickup electrodes (SP15-25 and SP16-25) newly installed at the $e^+$ capture section are described in Methods for technical details.

**Figure 1:**
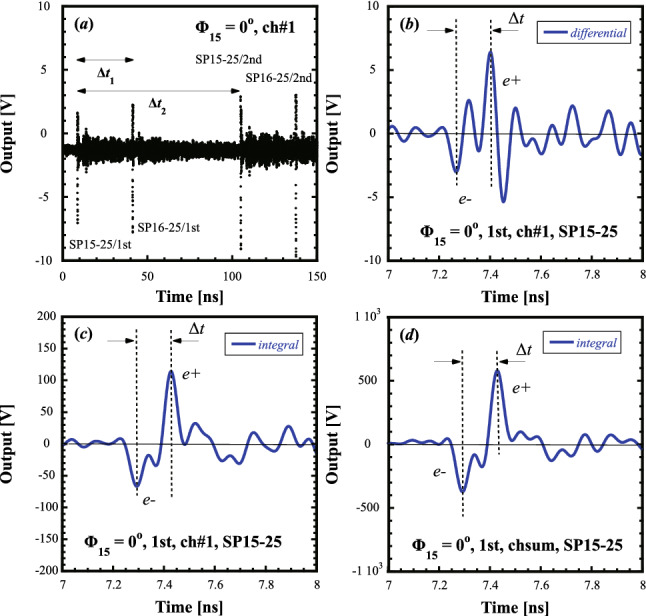
Typical detected pickup signals in the nominal $e^+$-production operation. (**a**) Corrected signal in the time span of 150 ns, (**b**) differential signal in the time span of 1 ns, (**c**) its integrated signal, (**d**) its integrated four-channel-sum signal. The detected signals in (**a**), (**b**), and (**c**) are from ch#1 of SP15-25 and that in (**d**) is from the four-channel sum at $\Phi _{15}=0^{\mathrm{o}}$.

The detected pickup signal of the WBM with four pickup electrodes (ch#1–ch#4) is a bipolar signal, which is a differential signal of a single-bunched beam in time. The typical pickup signals of SP15-25 in the nominal $e^+$-production operation are shown in Fig. 1.

Figure 1a shows the pickup signal (ch#1) of SP15-25 at $\Phi _{15}=0^\mathrm{o}$ after frequency-dependent amplitude-loss correction, where $\Phi _{15}$ indicates the capture phase of AC15. It can be seen that four successive signals are clearly detected in the time span of 150 ns. The former (later) two successive signals show that those of the first (second) bunch delivered in the two-bunch acceleration scheme are simultaneously detected by SP15-25 and SP16-25. Note that the two-bunch acceleration scheme means that two $e^{-}$ (or $e^{+}$) bunches are simultaneously accelerated in an *rf* pulse, in which the separation time between the two bunches is 96 ns. $\Delta t_1$ indicates the time duration due to the transit time between SP15-25 and SP16-25 and the time difference in the delay of the signal propagation to them, which is $\Delta t_1\simeq 32.3$ ns. On the other hand, $\Delta t_2$ indicates the time duration between the first and second bunches, which is $\Delta t_2\simeq 96.1$ ns. The peak-to-peak amplitudes for the four signals are all $\sim$13 V under the nominal operation condition. Note that the amplitude of the signal baseline before the first bunch measured by SP15-25 is due to intrinsic noise floor of the oscilloscope. On the other hand, the signal baseline level after the first bunch is due to the significant wakefields generated from upstream accelerating structures^7^.

Figure 1b shows an originally-detected differential signal (ch#1) of the first bunch of SP15-25, which is the expanded view of Fig. 1a in the time span of 1 ns. The differential signal comprises two successive bipolar signals. The former one shows the signal polarity (−, $+$) and the latter one indicates the signal polarity ($+$, −), which correspond to the $e^{-}$ and $e^{+}$ bunches in time sequence, respectively. Figure 1c shows the integrated waveform (ch#1) for this differential signal. The integrated waveform can be obtained by integrating waveform data of the differential signal in the horizontal time axis with carefully repeating subtraction procedures for the baseline offset. This signal again shows the former (latter) signal with minus (plus) polarity in time sequence, which clearly corresponds to the $e^{-}$ ($e^{+}$) bunch signal. It can easily be understood that the $e^{-}$ bunch precedes the $e^{+}$ bunch in time under this operation condition. The time interval $\Delta t$ between the peak positions of these bunch signals can clearly be seen from Fig. 1c.

There are still relatively significant baseline levels from the viewpoint of signal-to-noise ratio. Figure 1d shows the four-channel-sum signal (ch#1–ch#4) of the first bunch of SP15-25 to reduce the baseline level as much as possible. The sum signal is obtained from the signal sum after the peak positions of four channels for the $e^{-}$ bunch are made to coincide with each other in time. Note that it was verified that the time intervals $\Delta t$ analyzed in Fig. 1b–d were consistent with each other within measurement errors.

The result shows that the corrected signals are properly corrected for any transient responses up to the cutoff frequency, because any signal waveform distortion is suitably eliminated except for the baseline noise due to the significant wakefields. The oscilloscope can automatically correct the signals in the frequency domain and display them pulse-by-pulse in real time and in the time domain without any difficulty.

### Direct observation of the dynamical phase-slip process

**Figure 2:**
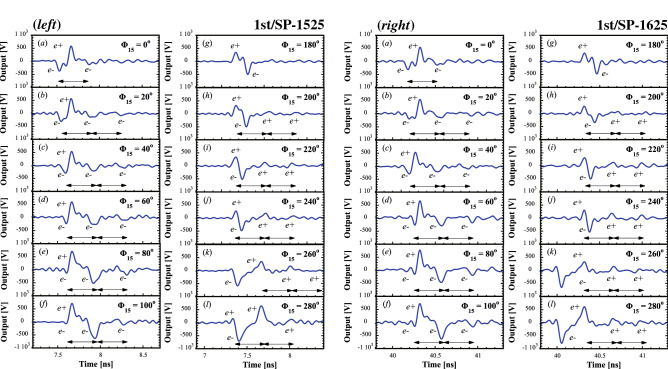
$({ left})$ Variations in the sum signal waveforms of WBM as a function of the capture phase $\Phi _{15}$ for the first bunch of SP15-25 ($\Phi _{15}=0^\mathrm{o}-100^\mathrm{o}$(**a**–**f**) and $\Phi _{15}=180^\mathrm{o}-280^\mathrm{o}$ (**g**–**l**). $({ right})$ Variations in the sum signal waveforms of WBM as a function of the capture phase $\Phi _{15}$ for the first bunch of SP16-25 ($\Phi _{15}=0^\mathrm{o}-100^\mathrm{o}$ (**a**–**f**) and $\Phi _{15}=180^\mathrm{o}-280^\mathrm{o}$ (**g**–**l**). The solid line arrows indicate a time scale of 350 ps.

Figure 2 shows the variation in the bunch configuration with time for the first bunch of $({ left})$ SP15-25 and $({ right})$ SP16-25 as a function of the capture phase $\Phi _{15}$ of AC15 with a step phase of 10$^{\mathrm{o}}$, for which the phase $\Phi _{16}$ of AC16 was concurrently changed by the same step phase. Here, note that the phases $\Phi _{15}=0^{\mathrm{o}}$ and $\Phi _{16}=0^{\mathrm{o}}$ are defined under the nominal operation condition.

It can be seen that at the nominal phase $\Phi _{15}=0^{\mathrm{o}}$, the $e^{-}$ bunch precedes the $e^{+}$ bunch in time sequence (see Fig. 2a). It is also found that a small amount of $e^{-}$ charges starts to be delayed from the $e^{-}$ main bunch, and $e^{-}$ satellite bunches are formed with a $\sim$350 ps delay from the main bunch. Here, a time scale of 350 ps indicated by a solid line arrow corresponds to one *rf* cycle of the fundamental accelerating frequency (2856 MHz), as shown in Fig. 2b. Note that the solid line arrows are drawn only in case that the satellite bunches are clearly identified. It is interesting that with increasing $\Phi _{15}$, the pulse areas of the $e^{-}$ satellite bunches increase along with that of the $e^{+}$ main bunch, whereas the pulse area of the $e^{-}$ main bunch decreases, as shown in Fig. 2c–f (called phase-slip process, see Methods for details). These results clearly show that the charges of both the main and satellite bunches vary in the phase-slip process since the bunch charge is proportional to the pulse area. At $\Phi _{15}=40^{\mathrm{o}}$, the pulse areas of the $e^{-}$ main and first satellite bunches are almost balanced, and the relationship between them is finally reversed at $\Phi _{15}=60^{\mathrm{o}}$. It can also be seen that the pulse widths of the $e^{-}$ main and satellite bunches vary as a function of the capture phase because the bunch length is defined as the pulse width. The results indicate that such behaviors of the bunches in the time sequence and the variations in the longitudinal asymmetric bunch shapes except for the nominal phase $\Phi _{15}=0^{\mathrm{o}}$ are still in the transient region in terms of the phase-slip process. The phase $\Phi _{15}=40^{\mathrm{o}}$ may be close to an intersection in time in terms of the interchange of the $e^{-}$ and $e^{+}$ main bunches. Here, note that only two $e^{-}$ satellite bunches can be detected with difficulty because the baseline fluctuation level due to wakefields limits their detection.

On the other hand, at $\Phi _{15}=180^{\mathrm{o}}$, the $e^{+}$ bunch precedes the $e^{-}$ bunch (see Fig. 2g). Figure 2g–l show the variations in the phase-slip process of the bunches in the phase regions between $\Phi _{15}=180^{\mathrm{o}}$ and $\Phi _{15}=280^{\mathrm{o}}$, which are 180$^{\mathrm{o}}$ apart from the previous $\Phi _{15}$ setting $(\Phi _{15}=0^\mathrm{o}-100^\mathrm{o})$. The results show that similar phase-slip processes can be found in which the entire reverse behaviors in the time sequence are generated when the $e^{-}$ and $e^{+}$ main bunches interchange their roles. It can be seen that the variations in the dynamical phase-slip process are almost the same at SP15-25 and SP16-25, because they arise mainly in the former accelerating region (AC15), and they are already fixed in the latter accelerating region (AC16).

**Figure 3:**
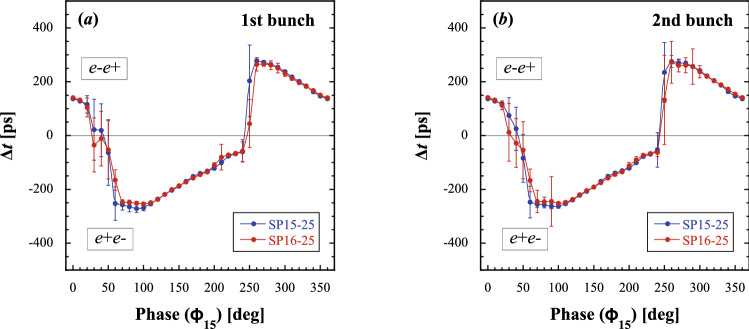
Variations in the time interval between the $e^{-}$ and $e^{+}$ (**a**) first and (**b**) second main bunches due to the dynamical phase-slip processes as a function of $\Phi _{15}$ at SP15-25 and SP16-25. The solid curves drawn through data are guide to the eye.

Figure 3 shows the variations in the time interval ($\Delta t$) between the $e^{-}$ and $e^{+}$ main bunches as a function of $\Phi _{15}$.

Figure 3a,b shows the variations in the time interval between the $e^{-}$ and $e^{+}$ first (second) main bunches due to the dynamical phase-slip processes as a function of $\Phi _{15}$ at SP15-25 and SP16-25. Each data point was repeatedly measured 100 times for averaging with an error of standard deviation. There are two intersections at $\Delta t=0$. One is at phase $\Phi _{15}\simeq 50^\mathrm{o}$ and the other is at phase $\Phi _{15}\simeq 230^\mathrm{o}$. The $e^{+}$ bunch precedes the $e^{-}$ bunch in the region of $\Phi _{15}=50^\mathrm{o}-230^\mathrm{o}$. The result shows that the $e^{+}$ ($e^{-}$) bunch is in the accelerating (decelerating) phase region, which corresponds to $\Delta t<0$ (see Methods for details). On the other hand, the $e^{+}$ ($e^{-}$) bunch is in the decelerating (accelerating) phase region, which corresponds to $\Delta t>0$, and also the phase regions of $\Phi _{15}=0^\mathrm{o}-50^\mathrm{o}$ and $\Phi _{15}=230^\mathrm{o}-360^\mathrm{o}$. It can be understood that the $e^{+}$ ($e^{-}$) bunch is in the decelerating (accelerating) phase region under the nominal operation condition. Note that the capture phase of $\Phi _{15}=50^\mathrm{o}$ ($\Phi _{15}=230^\mathrm{o}$) corresponds to a falling (rising) zero-cross phase of the fundamental accelerating *rf* wave.

The result shows that the dynamical capture process for the $e^{-}$ and $e^{+}$ bunches quite symmetrically proceeds in the $e^{+}$ capture section. The time intervals between the $e^-$ and $e^+$ first and second main bunches were measured in the range of 20–280 ps on average. It is interesting that the measurement error increases at the capture phases around the intersections. This is because the fluctuation in charge transfer between the main and satellite bunches based on the dynamical phase-slip process increases around the intersection phases. It can again be seen that the variations in the dynamical phase-slip process are almost the same at SP15-25 and SP16-25, because they arise mainly in the former accelerating region (AC15), and they are already fixed in the latter accelerating region (AC16).

**Figure 4:**
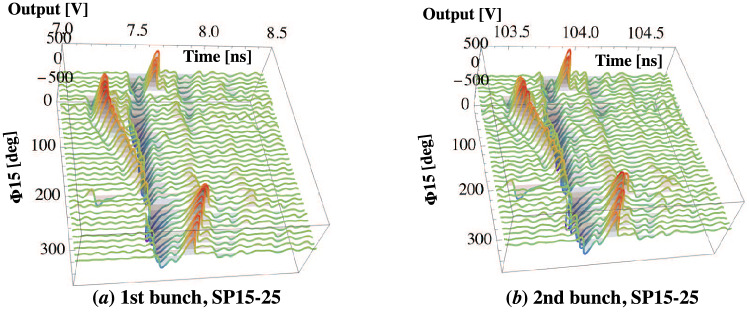
Three-dimensional (3D) plots for the $e^{-}$ and $e^{+}$ (**a**) first and (**b**) second main bunches as functions of the phase $\Phi _{15}$ and time at SP15-25. Note that the $e^{-}$ and $e^{+}$ main bunches are shown with the corresponding negative and positive output voltages, respectively. The reference time is given by the arriving time of the $e^{-}$ main bunch at SP15-25.

Figure 4a,b show the 3D plots as functions of $\Phi _{15}$ and time at SP15-25 for the $e^{-}$ and $e^{+}$ first and second main bunches, respectively, in which the reference time is fixed by the time at which the $e^{-}$ main bunch is detected at SP15-25.

It can be seen that not only the time interval between the $e^{-}$ and $e^{+}$ first main bunches but also the bunch charges and lengths clearly vary owing to complicated dynamical capture processes as functions of time and the capture phase $\Phi _{15}$. Similar behaviors in terms of such a dynamical capture process were successfully observed for both the $e^{-}$ and $e^{+}$ first and second main bunches at SP16-25.

## Conclusion

A direct simultaneous detection of the $e^-$ and $e^+$ bunches was successfully performed with the new WBMs and their detection system at the $e^+$ capture section of the SuperKEKB B-factory. The results show that the time intervals between the $e^-$ and $e^+$ first (also second) main bunches were measured in the range of 20–280 ps on average, and dynamical capture process for both the secondary-generated $e^-$ and $e^+$ bunches in the axial direction was clearly observed for the first time under the two-bunch acceleration scheme. Such wideband detection techniques can be applied to not only conventional $e^+$ sources but also advanced e+ sources in future accelerator projects. Since the obtained results may also improve simulations in any $e^+$ capture section, the $e^+$ intensity could systematically be optimized by applying this technique in a multidimensional beam parameter space towards high-intensity $e^+$ sources.

## Methods

### Positron source of the SuperKEKB B-factory

The SuperKEKB B-factory^8,9^ (SKEKB) is a next-generation B-factory that is currently in operation at KEK, after the KEKB B-factory^10,11^ (KEKB) was discontinued in 2010. The SKEKB is a $e^+e^-$ collider with asymmetric energies; it comprises 4 GeV $e^+$ (LER) and 7 GeV $e^-$ (HER) rings in which the designed stored beam currents are 3.6 A and 2.6 A, respectively. The target luminosity ($8 \times 10^{35}$ cm$^{-2}$s$^{-1}$) of the SKEKB, that is, the rate of $e^-$ and $e^+$ collisions, is 40 times the peak luminosity of the KEKB. The high-energy flavor particle physics experiments^12^, considering the CP violation in B mesons, are the main driver behind this study. To improve the collision rate, the development of a high-intensity and stable $e^+$ source is one of the key elements in this experiment.

**Figure 5:**
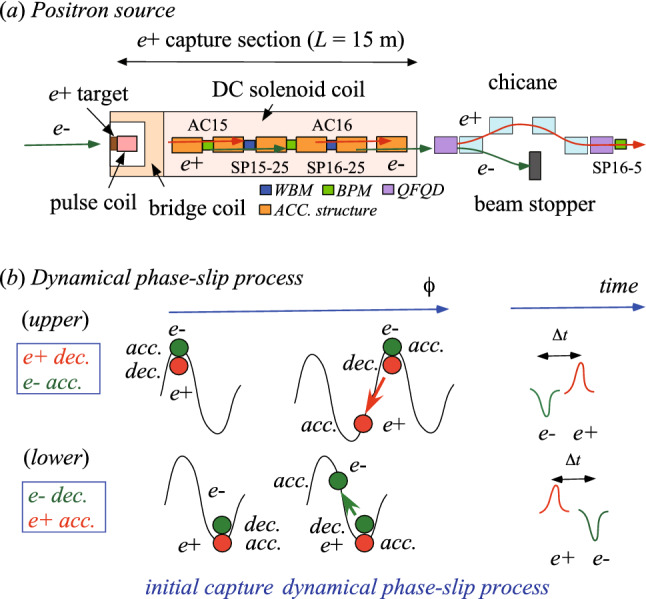
(**a**) Schematic drawing of the SKEKB $e^+$ source and (**b**) dynamical phase-slip process in the $e^+$ capture section.

The SKEKB injector linac^13^ is an $e^-e^+$ linear accelerator for the SKEKB; the KEKB injector linac^14^ was upgraded for the abovementioned purpose. The requirement for the injector linac is full energy injection into the SKEKB rings with the $e^-$ and $e^+$ bunch charges of 5 and 4 nC, respectively. The injector linac should deliver low-emittance and high-current $e^-$ and $e^+$ single-bunched beams to the SKEKB rings. The high-intensity $e^+$ beam is generated at the $e^+$ source, and they are to be damped to the level required for the low-emittance beam through a damping ring^15^. The SKEKB $e^+$ source is one of the conventional $e^+$ sources and its schematic drawing is shown in Fig. 5a. Other technical designs are described in more detail elsewhere^16^.

High-intensity positrons are generated by bombarding a tungsten target with high-energy primary electrons with an energy of 3.2 GeV and charges of 10 nC/bunch under the two-bunch acceleration scheme at a maximum repetition rate of 50 Hz, which can be delivered from a conventional thermionic $e^{-}$ gun. An off-axis $e^+$-production target ($e^{+}$ target) with 14-mm-thick tungsten was designed and built at the middle of sector 1 (unit 1-5). The off-axis target means that injection electrons pass through a 2-mm-diameter hole at the target center, whereas high-current primary electrons for $e^+$ generation hit the target at 3.5 mm off-axis in the horizontal direction from the target center. The primary and injection electrons can be switched by controlling the beam optics pulse-by-pulse with pulsed steering dipoles and quadrupole magnets installed in front of the target.

The generated positrons must be efficiently captured at the 15-m-long $e^+$ capture section. Immediately following the target, the capture section is located at units 1-5 and 1-6, which comprise magnetic matching devices based on AMD^9,16^ and accelerating structures. The accelerating structures comprise two 2-m-long large-aperture S-band accelerating structures^17^ (LAS, energy gain 14–20 MV/m) in unit 1-5 (AC15$\_$1 and $15\_$2) and four 2-m-long LAS (energy gain 10 MV/m) in unit 1-6 (AC16$\_$1–AC16$\_$4). They are powered by each high-power klystron with a SLED-type pulse compression system to boost the energy of the captured positrons to 120 MeV on average.

The large transverse emittances of the $e^+$ bunch emerging from the target are transformed to match the capture section aperture with its $\sim$0.5 T DC solenoids (SL, 0.4 T and 0.5 T in units 1-5 and 1-6, respectively) by a pseudoadiabatically changing solenoidal field consisting of a 3.5 T pulsed peak field from the FC as a strong $e^+$-focusing solenoid with a large energy acceptance. A bridge coil (BC) with a 1.5 T DC solenoidal field is also installed between the FC and downstream DC SLs to make the solenoidal field distribution smooth. The DC solenoidal field exists along the capture section.

A $e^{+}$ bunch can conventionally first be separated from an $e^{-}$ bunch at a chicane composed of four successive bending magnets. Quadrupole focusing and defocusing (QF and QD, respectively) magnetic systems are installed at both ends of the chicane. The electrons are stopped at the center of the chicane by a BS. The transverse positions and intensity of the $e^{+}$ bunch can be first measured with a BPM (SP16$\_$5) located after the chicane, since there have been no beam instrumentation devices in the capture section so far. The conversion factor defined by a number of positrons generated per primary $e^{-}$ (called $e^{+}$ yield) is experimentally investigated using this BPM to be $\sim$0.5 under the nominal operation condition.

Two conventional BPMs and two WBMs (SP15-25 and SP16-25) were newly installed in the $e^{+}$ capture section in the summer shutdown in 2020 ( see Fig. 5a). The SP15-25 (SP16-25) is located 5.1 m (5.2 m) behind the $e^{+}$ target (SP15-25). The BPMs are used to measure transverse positions and intensity of the high-energy injection $e^{-}$ beam. On the other hand, the WBMs with a bandwidth of $\sim$10 GHz are used to simultaneously measure transverse and longitudinal beam parameters of both the secondary $e^{+}$ and $e^{-}$ bunches generated at the target for $e^{+}$ production. The detailed technical design of the WBM was reported elsewhere^18,19^.

Figure  5b shows a schematic drawing of the dynamical phase-slip process for the secondary-generated $e^{-}$ and $e^{+}$ bunches in the $e^{+}$ capture section. Both the $e^{-}$ and $e^{+}$ bunches with low energies are almost simultaneously captured at an *rf* phase $\Phi _{15}$ of the first accelerating structure AC15 (called capture phase). When both the bunches are captured at an initial phase $\Phi _{15}=\pi /2$ (see Fig. 5b$-({ upper})$), the $e^{-}$ bunch is maximally accelerated, and on the other hand, the $e^{+}$ bunch is maximally decelerated by the longitudinal electric field in the accelerating structure. The $e^{-}$ bunch is accelerated to nearly the velocity of light (called $e^{-}$ bunch in accelerating phase region), and however, the $e^{+}$ bunch may be delayed with respect to the $e^{-}$ bunch (called $e^{+}$ bunch in decelerating phase region) due to the dynamical phase-slip process. After a finite time, both the bunches may reach to an equibrium state of their dynamical motion based on complicated beam dynamics with a time interval ($\Delta t$) between them.

When both the bunches are captured at an initial phase $\Phi _{15}=-\pi /2$ (see Fig. 5b$-({ lower}))$, the similar phase-slip process may be reproduced in which the entire reverse behaviors in the time sequence are generated when the $e^{-}$ and $e^{+}$ main bunches interchange their roles. The time interval may be almost determined by the capture phase of AC15. Thus, the line order of the $e^{-}$ and $e^{+}$ bunches and their time interval are transiently changed and measured at the locations of WBMs.

### Simultaneous detection system of $e^+$ and $e^-$ bunches at the $e^+$ capture section

**Figure 6:**
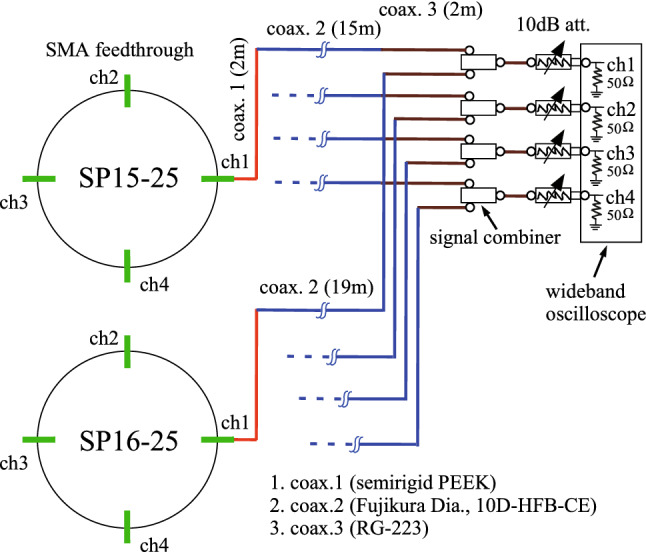
Simultaneous detection system for the two WBMs.

The signal detection system has already been reported in detail elsewhere^18,20^. It is briefly summarized here. Figure 6 shows the signal detection system for the two WBMs. The pickups of the WBM are made of SMA-type vacuum feedthroughs composed of a central conductor pin made of Kovar and a dielectric substance made of ceramic. The four pickups, two horizontal and two vertical, are mounted on the WBM with $\pi /2$ rotational symmetry. The WBM can measure longitudinal and also transverse beam parameters thanks to the four pickups. The tips of the center pins protrude for a length of 1 mm toward the monitor center from the inner surface of the monitor.

Two sets of four signals detected with high-frequency pickups from each WBM with an aperture diameter of 38 mm are sent to four *rf* combiners (Marki Microwave, PD-0R618, BW 0.6–18 GHz^21^) with coaxial cables, where the eight signals are combined. A set of the combined four signals are directly sent to a wideband real-time oscilloscope (Keysight Technologies, Infiniium V DSOV134A, BW 13 GHz, 40 GSa/s^22^). The *rf* combiners are independently connected to four input channels of the oscilloscope with a fixed attenuator of 10 dB in front of each input channel, which is terminated with an internal 50-$\Omega$ terminator. The signal waveforms should be measured under proper conditions with corrections on the frequency-dependent amplitude losses of the transmission cables and *rf* combiners. These losses can be corrected in frequency domain in the oscilloscope by applying a standard fast-Fourier transformation (FFT)^23^. The losses were measured with a vector network analyzer in advance. The signal waveforms corrected in frequency domain can be displayed pulse-by-pulse in the time domain by applying an inverse FFT using a software-based Bessel filter with fourth-order roll-off at the cutoff frequency of 9.1 GHz (see Ref. ^18^ for details).

## Data Availability

The datasets used and/or analysed during the current study available from the corresponding author on reasonable request.
